# Nonwearable Gaze Tracking System for Controlling Home Appliance

**DOI:** 10.1155/2014/303670

**Published:** 2014-09-14

**Authors:** Hwan Heo, Jong Man Lee, Dongwook Jung, Ji Woo Lee, Kang Ryoung Park

**Affiliations:** Division of Electronics and Electrical Engineering, Dongguk University, Seoul 100-715, Republic of Korea

## Abstract

A novel gaze tracking system for controlling home appliances in 3D space is proposed in this study. Our research is novel in the following four ways. First, we propose a nonwearable gaze tracking system containing frontal viewing and eye tracking cameras. Second, our system includes three modes: navigation (for moving the wheelchair depending on the direction of gaze movement), selection (for selecting a specific appliance by gaze estimation), and manipulation (for controlling the selected appliance by gazing at the control panel). The modes can be changed by closing eyes during a specific time period or gazing. Third, in the navigation mode, the signal for moving the wheelchair can be triggered according to the direction of gaze movement. Fourth, after a specific home appliance is selected by gazing at it for more than predetermined time period, a control panel with 3 × 2 menu is displayed on laptop computer below the gaze tracking system for manipulation. The user gazes at one of the menu options for a specific time period, which can be manually adjusted according to the user, and the signal for controlling the home appliance can be triggered. The proposed method is shown to have high detection accuracy through a series of experiments.

## 1. Introduction

The rapid development of technology has led to considerable research on human-computer interaction to help handicapped people. Mauri et al. introduced computer-assistive technology for interaction with personal computers via devices such as switches, joysticks, trackballs, head pointers, neural interfaces, and eye tracking systems [1]. Switches, joysticks, trackballs, and head pointers are widely used as computer-assistive devices. They can be activated by hands, feet, chin, mouth, thumbs, palm, or head, as well as by blowing, and can be used as a mouse pointer or for controlling a wheelchair [1]. Computer vision-based head and face tracking also enables the control of mouse pointers [1–3]. However, these methods cannot be easily used by people with severe disabilities, such as quadriplegia, a condition in which a person cannot move hands, feet, and head.

Patients with severe motor disabilities can use different bioelectrical signals such as electroencephalograms (EEGs), electromyograms (EMGs), and electrooculograms (EOGs) for communication [4]. The EEG signal, which is based on cerebral waves, can be used to control a virtual keyboard, mouse, or wheelchair [4, 5]. Similarly, EMG signals, which are based on muscle responses, can also be used to interact with other systems [4, 6]. The EOG signal can be used for simple interaction because it determines the approximate gaze direction depending on eye movements [4, 7, 8]. However, the device that measures EOG signals is expensive, and attaching the EOG sensor around the eye is uncomfortable. Therefore, camera-based gaze tracking methods have been extensively researched. The eye gaze tracking methods reported previously in [9–11] used 2D monitors of desktop computers. However, these methods have some drawbacks. For example, the variation in the distance between a user and monitor, referred to as *Z*-distance henceforth, is limited whereas different *Z*-distances exist between a user and different home appliances in practice. In addition, the calibration positions can be indicated in a monitor in case of the gaze estimation in the 2D monitor. However, we cannot show the calibration positions in the monitor because the user can gaze at the home appliance which does not have monitor.

To overcome these limitations, we propose a novel nonwearable gaze tracking system for controlling home appliances in 3D space. The proposed system can also be used by severely disabled people. Because our system enables a user to control home appliances by looking at them rather than by gazing at a menu on a display, it is more natural and convenient for users. This is because the method that involves gazing at a menu must also include a complicated menu layout.

Existing gaze tracking method based assistive technologies can be categorized as those using a 2D screen and those operating in 3D space. The first category technologies use gaze-estimation information to control the cursor on a 2D screen [9–15]. This method uses mapping between the fixed screen region and the corresponding gaze region. In [9, 10], a wearable eye tracking system on a head-mounted device is used to control a wheelchair and a mouse. A nonwearable gaze tracking system based on a visible light web camera is proposed in [11]. In this system, a face is detected on the basis of skin color, and the eye is tracked to control the mouse on the basis of projections of edge, luminance, and chrominance. Magee et al. proposed an eye gaze tracking method for controlling applications such as spelling programs and games [12]. An artificial neural network has been used to determine the relationship between the gaze coordinates and mouse position, thus minimizing mouse jitter caused by saccadic eye movements [13]. Lee et al. proposed a remote gaze detection method with wide- and narrow-view cameras that have panning and tilting functionalities and used this system as an interface for intelligent TVs [14]. In [15], a system using a head-mounted device with two cameras for eye tracking and frontal viewing has been proposed. This system can be used by cerebral palsy patients to communicate with others by selecting symbols on communication boards on a 2D display.

The second category technologies utilize gaze estimation information to control an object in 3D space [16–21]. Shi et al. proposed a gaze detection system that contains two cameras for eye tracking and frontal object detection [16]. However, they used the scale invariant feature transform (SIFT) method to recognize the frontal viewing object, thus resulting in a limited *Z*-distance range (where the object can be recognized) and a high processing time. Further, the recognition accuracy is degraded if the size of the object is small with a large *Z*-distance between the camera and object. In addition, when a TV is turned on, different images are displayed on the TV screen, and they cannot be easily recognized by the SIFT algorithm. Moreover, their method requires a user to set up an additional target chart with nine calibration points and gaze at these calibration points for initial calibration, which requires considerable processing time and is inconvenient for the user.

In the previous methods [17–19], a head-mounted display (HMD) system (of helmet or glasses type) must be worn for an extended period of time, which is inconvenient and tiring. In [17], an HMD system with two cameras for eye gaze tracking and frontal viewing was proposed for augmented reality. However, object recognition in the image captured by the frontal viewing camera must be performed using a specific marker pattern. In addition, wearing the HMD over long periods of time can be inconvenient and may cause cybersickness. Mardanbegi and Hansen proposed a method for users to interact with multiple screens via an HMD containing cameras for eye tracking and frontal viewing [18]. However, their method for identifying the screen in the frontal viewing image is based on the detection of edge contours; therefore, its applicability is limited in cases where the screen has a complex background. The method in [19] employs an HMD with one camera for eye gaze tracking and two scene cameras for frontal viewing. By using two scene cameras, the viewing range can be increased to match that of natural head movements of a user. However, no method was proposed for recognition of an object in the scene camera image. Weibel et al. developed a mobile eye tracking system for observational research on flight decks [20]. However, they used the template matching-based method to identify objects in images captured by the frontal viewing camera. This limits the *Z*-distance range and the viewing angle from which an object can be recognized because a pilot uses this system while sitting in the cockpit. Hales et al. proposed a wearable device consisting of a camera for eye gaze tracking and one for object and gesture recognition in the frontal viewing image [21]. However, the visible marker based method for object recognition requires a long processing time and has a limited *Z*-distance range in which the marker can be recognized.

Majaranta and Räihä presented the features, functionalities, and methods of previous eye typing systems based on eye tracking. In addition, they proposed a communication method concerned with text production [22]. Lankford presented the different methods for executing the mouse actions of full ranges and for eye-based typing [23]. They used the eye gaze response interface computer aid (ERICA) system by the University of Virginia for experiments. Jacob and Karn presented the different methods of applying eye movements to user interface (UI) in order to analyze interfaces considering usability and control within a human-computer dialogue. Previously, these two issues were dealt with separately; Jacob and Karn combined them in [24]. However, [22–24] primarily deal with gaze-based UIs for 2D computer monitors, which is different from the present research of selecting home appliances in 3D space through gaze tracking.

Therefore, we propose a new nonwearable gaze tracking system for controlling home appliances in 3D space using two near-infrared (NIR) cameras for gaze tracking and frontal viewing. By attaching multiple NIR light-emitting diodes (LEDs) to their outer boundaries, different home appliances, in different positions, can be easily recognized by the NIR frontal viewing camera. A simple user calibration process in which the user gazes at the four corners of a home appliance enables the detection of the final gaze position toward the appliance.  Table 1 shows the summary of the proposed and existing methods.

The structure of this paper is as follows. The proposed system and our methodology are presented in Section 2.  Section 3 describes the experimental setup and the results, and the conclusions and some ideas for future work are presented in Section 4.

## 2. Proposed Device and Methods

**Figure 1:**
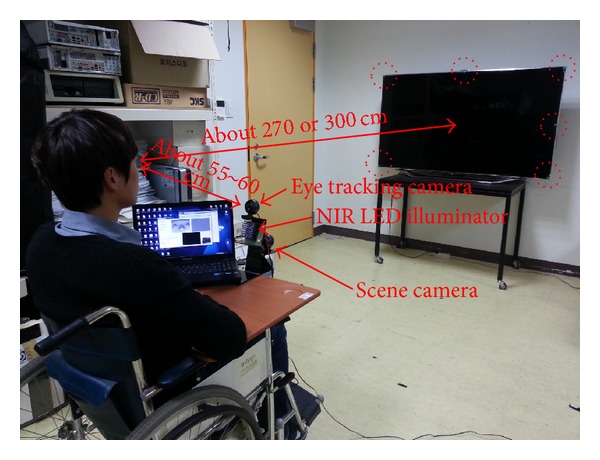
Proposed system.

Figure 1 shows a photograph of the proposed device with the eye tracking and scene cameras and an NIR illuminator. These are the nonwearable devices attached to the wheelchair. A Logitech C600 commercial web camera with a universal serial bus (USB) interface is used for the eye tracking and scene cameras [25]. The NIR cutting filter inside the camera is replaced by an NIR passing filter to ensure that the images captured by the eye tracking and scene cameras are not affected by the exterior lighting conditions. The accuracy of gaze estimation is typically higher with high-resolution eye images than that with low-resolution eye images. Therefore, the eye tracking camera has a resolution of 1600 × 1200 pixels. Because the accuracy of detecting NIR LED spots in a scene image is minimally affected by image resolution, the scene camera produces images of 640 × 480 pixels, considering the bandwidth limit of data transfer in a USB 2.0 web camera. Considering the synchronization of images of the eye tracking and scene cameras, we use images acquired at approximately 10 fps for our system. The eye camera is positioned below the eye to prevent obstruction of line of sight of the user at a *Z*-distance of approximately 55 cm–60 cm. To obtain a large eye image at this *Z*-distance, the eye camera is equipped with a zoom lens. The illuminator of 8 × 8 NIR LEDs is attached below the eye tracking camera, as shown in Figure 1. The wavelength of the NIR LEDs is approximately 850 nm, which does not dazzle the eye of the user but provides a distinctive boundary between the pupil and the iris. The dotted circles in Figure 1 indicate the NIR LEDs.

**Figure 2:**
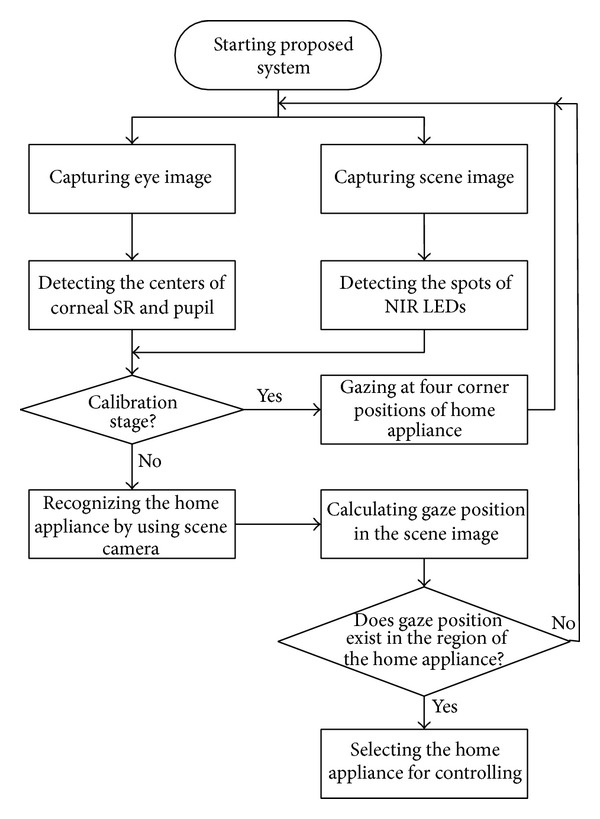
Flowchart of the proposed system.

Figure 2 shows the operation flowchart of the proposed system. After the system is turned on, the nonwearable eye tracking and scene cameras on the wheelchair capture the eye and scene images, respectively. In the eye image, the centers of the corneal specular reflection (SR) and the pupil are detected, as explained in Section 2.1. In the scene image, the spots of the NIR LEDs are located, as explained in Section 2.2. In the initial calibration stage, the user gazes at the four corners of the home appliance to obtain the mapping matrix between the pupil's movable region in the eye image and the object region in the scene image (Section 2.3). The home appliance is recognized by the scene camera on the basis of the number of NIR LED spots and their patterns (Section 2.2). The user's gaze position is then calculated using the mapping matrix in the scene image (Section 2.4). If the gaze position is located in the region of a home appliance in the scene image, the system selects that home appliance for control. Even if the gaze position is located at the corner or on the boundary of the home appliance region in the scene image, the proposed system selects the home appliance to be controlled.

### 2.1. Detecting Corneal SR and Pupil

**Figure 3:**
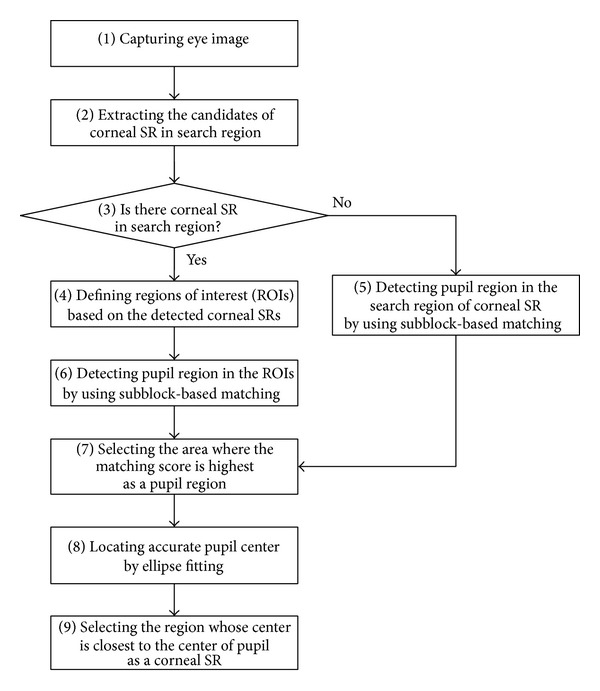
Flowchart for detection of corneal SR and pupil regions.

After an image is captured by the eye tracking camera, the corneal SR and pupil are detected, as shown in Figure 3.

**Figure 4:**
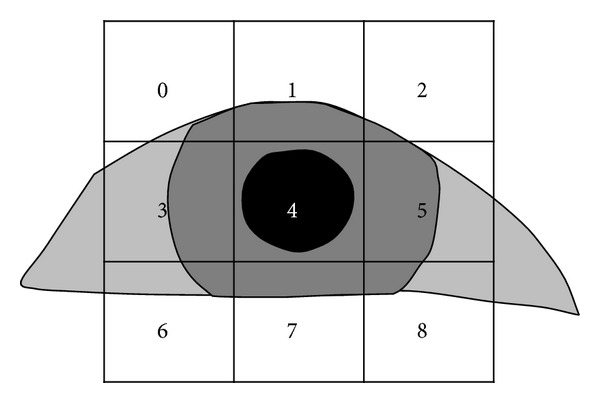
Example of subblock-based matching for pupil detection.

Figure 3 shows the flowchart for the detection of the corneal SR and pupil regions. After the eye tracking camera captures an image, the candidate corneal SRs in a predefined search region are extracted using image binarization, component labeling, and size filtering [26]. The regions of interest (ROIs) for detecting the pupil are then defined depending on the detected corneal SR candidates, and the approximate position of the pupil is determined using subblock-based matching. In the subblock-based matching process, nine subblocks are defined, and the position at which the difference between the mean of the central subblock (4 in Figure 4) and the surrounding subblocks is maximized is determined to be the pupil region [27]. This procedure is repeated by moving the 3 × 3 mask, with these nine subblocks, in the ROIs. The integral imaging method is used to reduce the computational complexity by calculating the average intensity of each subblock [27].

**Figure 5:**
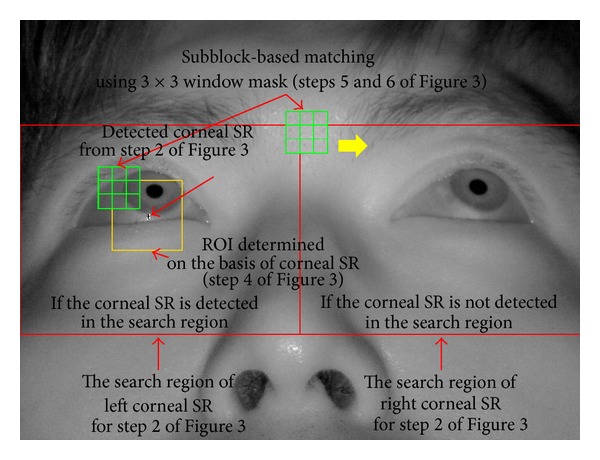
Sample detection of the corneal SR and approximate pupil regions.

**Figure 6:**
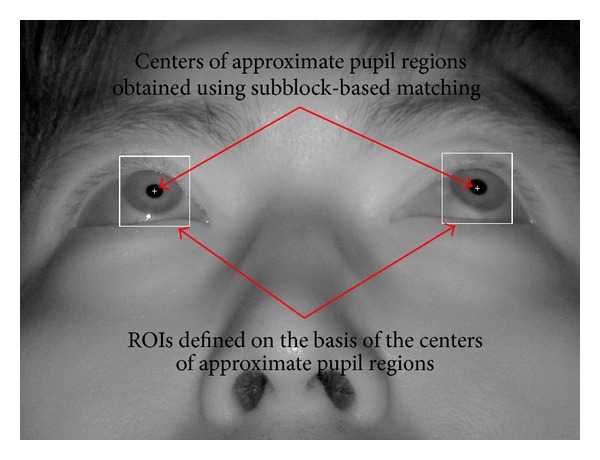
Result of the sample pupil region detection.

If no corneal SR is detected in the search region, the subblock-based matching process is performed in this area. The subblock-based matching method uses different subblock sizes (from 20 × 20 pixels to 60 × 60 pixels) depending on the pupil size, which is affected by variations in illumination and the *Z*-distance between the camera and user's eye.  Figure 5 shows an example of detecting the corneal SR and approximate pupil regions.  Figure 6 shows the result of sample pupil detection.

**Figure 7:**
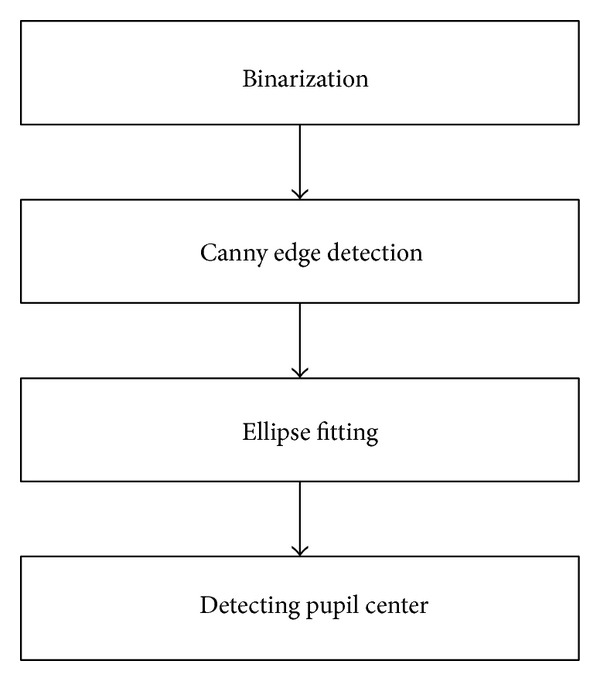
Flowchart for the accurate detection of pupil centers.

The pupil region shown in Figure 6 is the approximate position of the pupil. An accurate estimate of the center of the pupil is required to accurately determine the gaze position. For detecting the accurate pupil center, an ROI is defined on the basis of the center of the approximate pupil region, as shown in Figure 6. Within this ROI, the pupil center is accurately located using the procedure shown in Figure 7.

**Figure 8:**
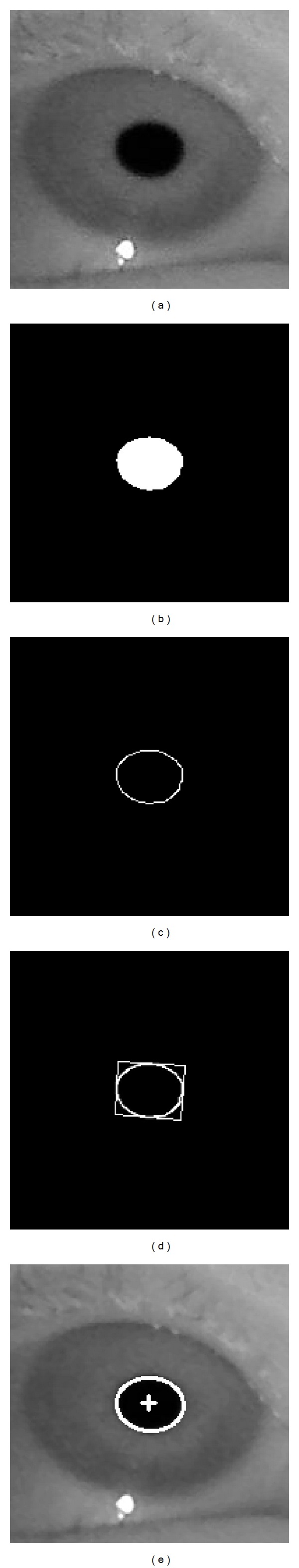
The procedure of accurately detecting pupil centers. (a) Original image. (b) Binarization image of (a). (c) Canny edge detection image of (b). (d) Ellipse fitting image of (c). (e) Image of detected pupil center.

Figure 7 shows the flowchart of the accurate pupil center detection method. First, an image binarization is performed on the basis of the ROI shown in Figure 6, as shown in Figure 8(b). The threshold value for image binarization is determined using Gonzalez's method [28]. Canny edge detection is performed to locate the edge of the pupil region [29], as shown in Figure 8(c). The pupil center is then detected with an ellipse fitting algorithm, as shown in Figure 8(d). The final result of the pupil center detection is shown in Figure 8(e).

The primary differences between the proposed and the other video-based combined pupil/corneal reflection methods are as follows. In our research, the ROI for eye detection is significantly reduced by the subblock-based template matching algorithm within the left (or right) corneal SR search region (as shown in Figure 5) for high accuracy and fast processing of eye detection. In addition, the accurate pupil center is located via ellipse fitting, as shown in Figure 7.

### 2.2. Recognizing Home Appliances Using Frontal Viewing Camera

**Figure 9:**
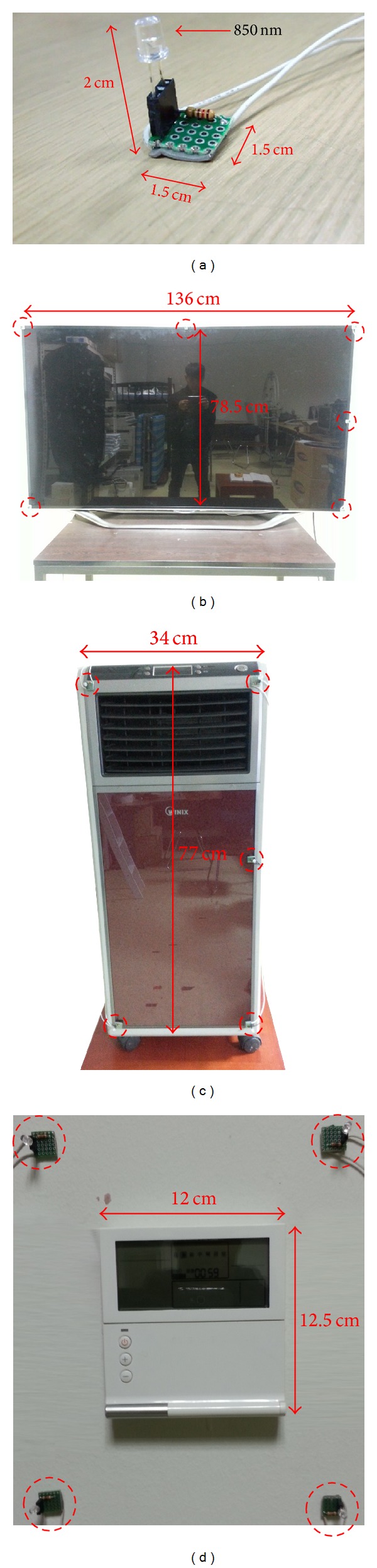
(a) An NIR LED. Attachment of NIR LEDs on home appliances such as (b) a 60-inch television (six NIR LEDs), (c) a heater (five NIR LEDs), and (d) an air conditioner switch (four NIR LEDs). Note that the dotted circles indicate the NIR LEDs.

In the proposed system, home appliances such as TVs, heaters, and air conditioner switches are recognized by the scene camera shown in Figure 1. In the images captured by the scene camera, the shape of the home appliances may vary depending on the position of the scene camera. In addition, they may also change with illumination variations. If the TV is turned on, different shapes may be produced by the program running on the TV. Therefore, recognition of every home appliance is very difficult. To overcome these problems, multiple NIR LEDs are attached at the outer boundaries of the home appliances (the red dotted circles of Figures 9(b)–9(d)), and an NIR scene camera is employed. The camera is robust to different conditions such as variations in exterior lighting conditions, object size, and viewpoints. The wavelength of the NIR LEDs is 850 nm.

To allow user-dependent calibration for eye tracking (Section 2.3), four NIR LEDs are attached at the four corners of home appliances, as shown in Figures 9(b)–9(d). Because the air conditioner switch is too small to be recognized by the scene camera, the NIR LEDs are attached at positions slightly outside the four corners of the switch, as shown in Figure 9(d).

**Figure 10:**
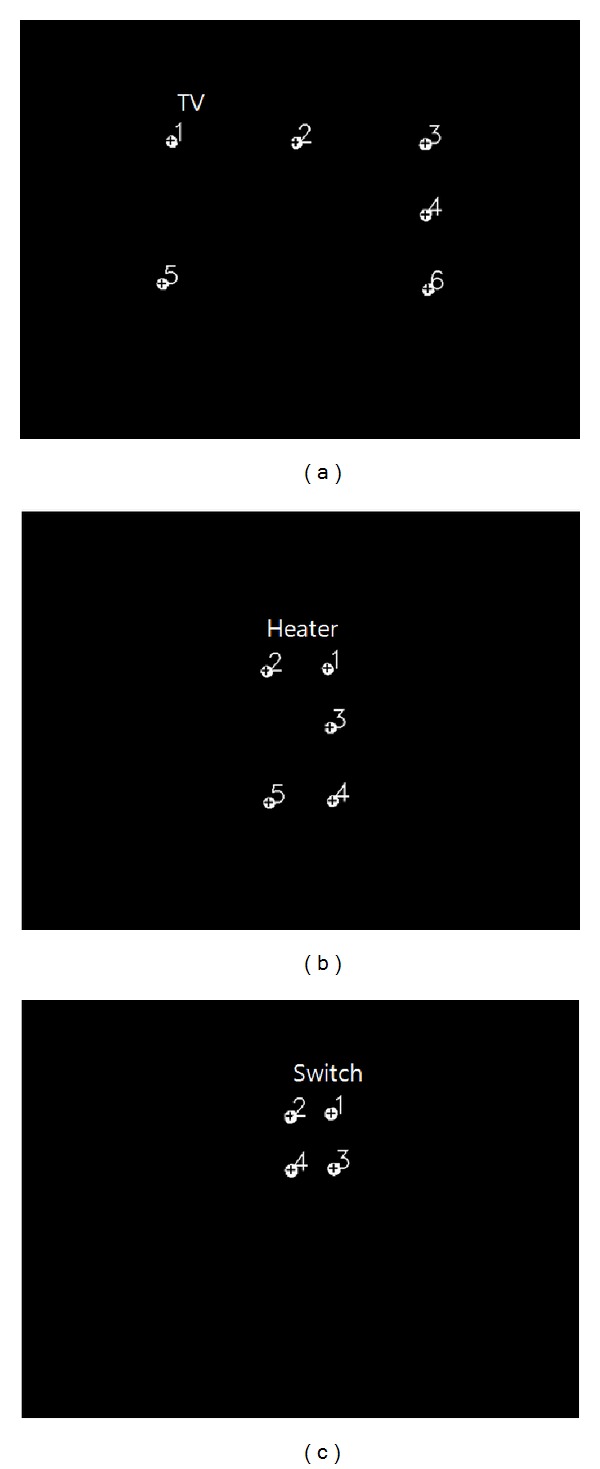
Results of recognition of home appliance in (a) Figure 9(b) at a *Z*-distance of 300 cm, (b) Figure 9(c) at a *Z*-distance of 300 cm, and (c) Figure 9(d) at a *Z*-distance of 200 cm.

Therefore, the home appliances can be recognized on basis of the patterns and the number of NIR LEDs detected by the NIR scene camera, as shown in Figure 10. The specifications of the NIR LEDs and NIR camera indicate that only the spots of the NIR-LEDs can be seen in the camera image. Thereafter, the NIR LEDs in the image captured by the scene camera can be easily extracted by binarization and component labeling. Depending on their quantity and the pattern of the spots, the home appliances can then be recognized, as shown in Figure 10. Different patterns can be generated with the NIR LEDs; thus, different home appliances can be recognized. The proposed recognition method using NIR LEDs and an NIR camera is robust to variations in exterior lighting conditions, object size, and viewpoints and has a fast computation speed. In Figure 10, the *Z*-distance is the distance between the user's eye and the home appliance.

### 2.3. Initial User-Dependent Calibration

**Figure 11:**
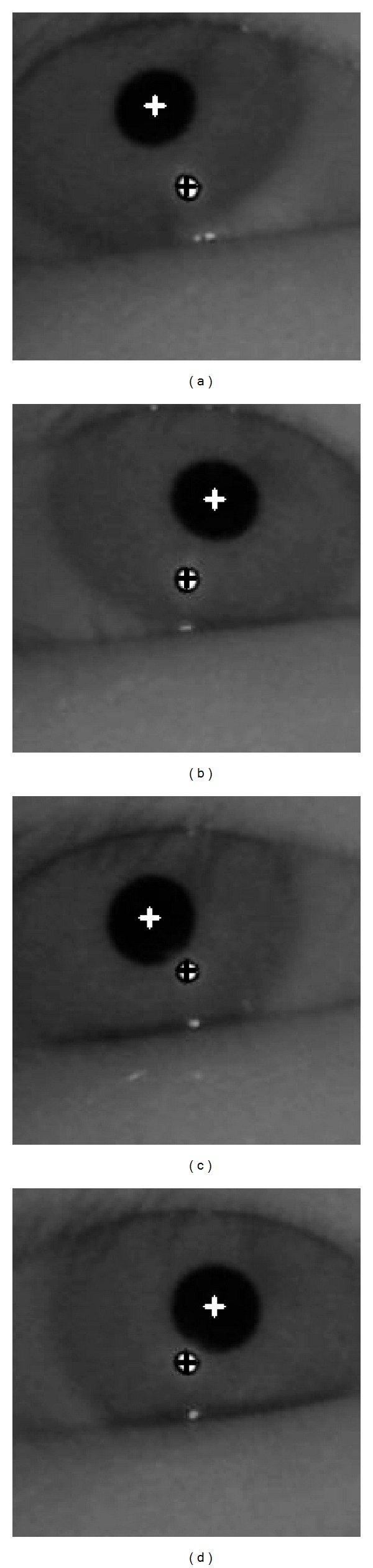
Images of the detected centers of pupil and corneal SR when a user is gazing at the (a) upper left-hand, (b) upper right-hand, (c) lower left-hand, and (d) lower right-hand corners of the 60-inch television.

To calculate the gaze position in the scene image, an initial user-dependent calibration must be performed, as shown in Figure 11. For the calibration, the user gazes at the four (upper left-hand, upper right-hand, lower right-hand, and lower left-hand) corners of the home appliances at which the NIR LEDs are attached, as shown in Figures 9(b)–9(d).

Using this calibration procedure, the four center positions of the pupil and corneal SR can be obtained, as shown in Figure 11. The corneal SR positions are used to compensate for head movement when gazing at a point on a home appliance. In each image, the position of the corneal SR is first set to the same position, and the position of the pupil center is moved by the amount of movement in the corneal SR. Namely, the position of the pupil center is compensated by ensuring that the corneal SR position is the same in each image. For example, the positions of the pupil center and corneal SR are (100, 100) and (120, 120), respectively, in the first image. In addition, those of the pupil center and corneal SR are (130, 90) and (110, 130), respectively, in the second image. The deviations between the two corneal SR positions are −10 (110−120) and +10 (130−120) on the *x*- and *y*-axis, respectively. Therefore, if we try to set the corneal SR position of the first image (120, 120) to be the same as that of the second image (110, 130), the pupil center position of the first image (100, 100) is (90, 110) on the basis of the disparities (−10, +10).

If no corneal SR is detected, as in the case of right eyes of Figures 5 and 6, the original pupil center positions are used for calibration and gaze estimation without compensation. In this case, the original pupil center represents the uncompensated position (by the corneal SR position), which is detected using ellipse fitting, as shown in Figure 7. In the above example, if the corneal SR is not detected in the first image, the pupil center position of (100, 100) is used for calculating the gaze position.

### 2.4. Calculation of Gaze Position on Scene Image

**Figure 12:**
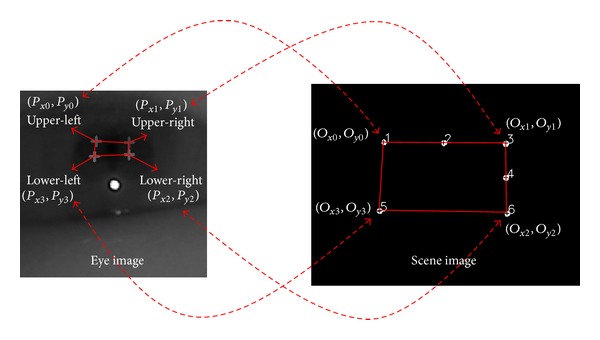
Mapping relationship between the pupil movable region (the rectangle given by (*P*_*x*0_, *P*_*y*0_), (*P*_*x*1_, *P*_*y*1_), (*P*_*x*2_, *P*_*y*2_), and (*P*_*x*3_, *P*_*y*3_)) on the eye image and the recognized region of the home appliance (the rectangle given by (*O*_*x*0_, *O*_*y*0_), (*O*_*x*1_, *O*_*y*1_), (*O*_*x*2_, *O*_*y*2_), and (*O*_*x*3_, *O*_*y*3_)) on the scene image.

The following four pupil center positions are obtained in the initial calibration stage from Figure 11: (*P*_*x*0_, *P*_*y*0_), (*P*_*x*1_, *P*_*y*1_), (*P*_*x*2_, *P*_*y*2_), and (*P*_*x*3_, *P*_*y*3_) (see left-hand side image of Figure 12). The user actually gazes at the four corners of the home appliance, and these positions are observed in the scene camera image as (*O*_*x*0_, *O*_*y*0_), (*O*_*x*1_, *O*_*y*1_), (*O*_*x*2_, *O*_*y*2_), and (*O*_*x*3_, *O*_*y*3_) (see right-hand side image of Figure 12). Hence, the relationship between the rectangles given by (*P*_*x*0_, *P*_*y*0_), (*P*_*x*1_, *P*_*y*1_), (*P*_*x*2_, *P*_*y*2_), and (*P*_*x*3_, *P*_*y*3_) and (*O*_*x*0_, *O*_*y*0_), (*O*_*x*1_, *O*_*y*1_), (*O*_*x*2_, *O*_*y*2_), and (*O*_*x*3_, *O*_*y*3_)  can be given by a geometric transform matrix, as shown in (1). The final gaze position can be calculated using this matrix and the detected pupil and corneal SR centers, as given by (2) [14, 30, 31].

As shown in Figure 12, the relationship between the two rectangles can be mapped using a geometric transform. The equations for this geometric transform are given as follows [14, 30, 31]:

(1) [Ox0Ox1Ox2Ox3Oy0Oy1Oy2Oy300000000] =[abcdefgh00000000][Px0Px1Px2Px3Py0Py1  Py2  Py3Px0Py0Px1Py1Px2Py2    Px3Py31111].

The matrix coefficients *a*–*h* can be calculated from (1). Using these matrix coefficients, one pupil position (*P*_*x*_′, *P*_*y*_′) is mapped to a gaze position (*G*_*x*_, *G*_*y*_) on the scene image as follows [14, 30, 31]:

(2) $$\begin{array}{c} \left[\begin{array}{c} G_{x}\\ G_{y}\\ 0\\ 0 \end{array}\right]=\left[\begin{array}{cccc} a & b & c & d\\ e & f & g & h\\ 0 & 0 & 0 & 0\\ 0 & 0 & 0 & 0 \end{array}\right]\left[\begin{array}{c} P_{x}^{\prime }\\ P_{y}^{\prime }\\ P_{x}^{\prime }P_{y}^{\prime }\\ 1 \end{array}\right]. \end{array}$$

**Figure 13:**
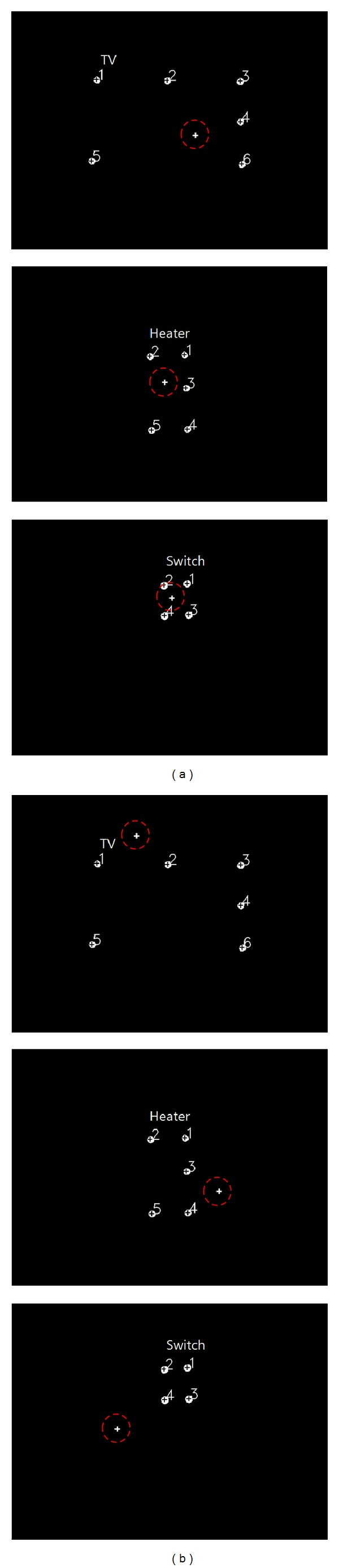
Examples of (a) selection and (b) nonselection of a home appliance by gaze estimation. Note that the red dotted circles indicate the calculated gaze position.

In the proposed system, the average gaze position of the left and right eyes is considered to be the final gaze position. If the final gaze position is within the region of the home appliance, as shown in Figure 13(a), for a predetermined time period (2 s), the system determines that the user wants to select the corresponding home appliance for control. The home appliance area is the rectangular region defined by the four spots of the NIR LEDs attached at the four corners. The dwell time (2 s) is experimentally determined considering the user's preference.

## 3. Experimental Results

The performance of the proposed system was evaluated with two experiments. The gaze estimation uncertainty was analyzed in the first experiment, and the accuracy with which home appliances are selected was determined in the second experiment. A laptop computer (Intel Core i5 at 2.5 GHz with 4 GB of memory) was used for both experiments. The proposed method was implemented using the OpenCV Microsoft Foundation Class (MFC) library [32].

### 3.1. Gaze Estimation Uncertainty

**Figure 14:**
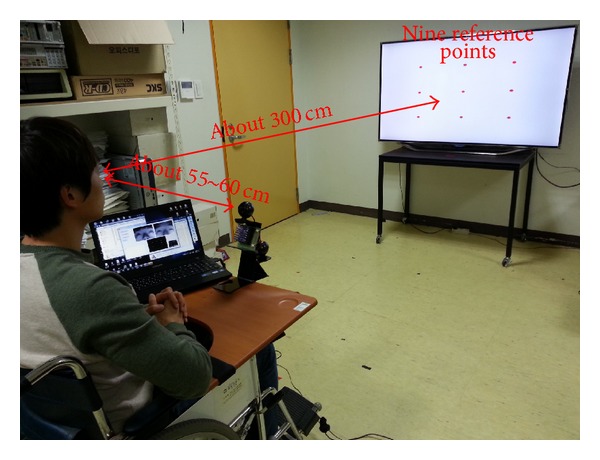
Experimental setup for measuring gaze estimation error.

To calculate the gaze estimation uncertainty (the error of gaze estimation) in our system, 10 users were asked to gaze at nine reference points on a 60-inch television. This procedure was repeated five times. The diameter of each reference point is 2 cm, and the *Z*-distance between the users' eyes and the television was approximately 300 cm. Because only the gaze estimation uncertainty and not the accuracy of home appliance selection is determined in this experiment, only the television was used, as shown in Figure 14. In the initial user calibration stage, each user was asked to gaze at the four NIR LEDs attached at the four corners of the television. So, the gaze estimation uncertainty was then measured when each user gazed at the nine reference positions (of Figure 14), which are not used for user calibration and are not biased to the calibration information. Therefore, the gaze estimation uncertainty can be measured more accurately. Figure 14 shows the experimental setup used for measuring the gaze estimation error.

The gaze estimation uncertainties (error) were calculated from the difference between the calculated and the reference gaze positions, as shown in Table 2. The average error is approximately ±1.04°.

The gaze estimation uncertainty for the subjects ranges from 0.51° to 2.06°, shown in Table 2, depending on the accuracy of user calibration. For instance, user 8 correctly gazed at the four calibration positions (the four NIR LEDs attached at the four corners of the television) in the initial user calibration stage whereas users 4 and 5 did not.

### 3.2. Accuracy of Home Appliance Selection

To measure the accuracy with which home appliances can be selected, experiments were conducted for two cases: users gazing and not gazing at a home appliance. A total of 20 people participated in the experiments. Among them, 18 were male and 2 were female, and none of them wore glasses or contact lens. The average age (standard deviation) of the 20 participants is 27.4 (1.85), and all of them are able-bodied.

**Figure 15:**
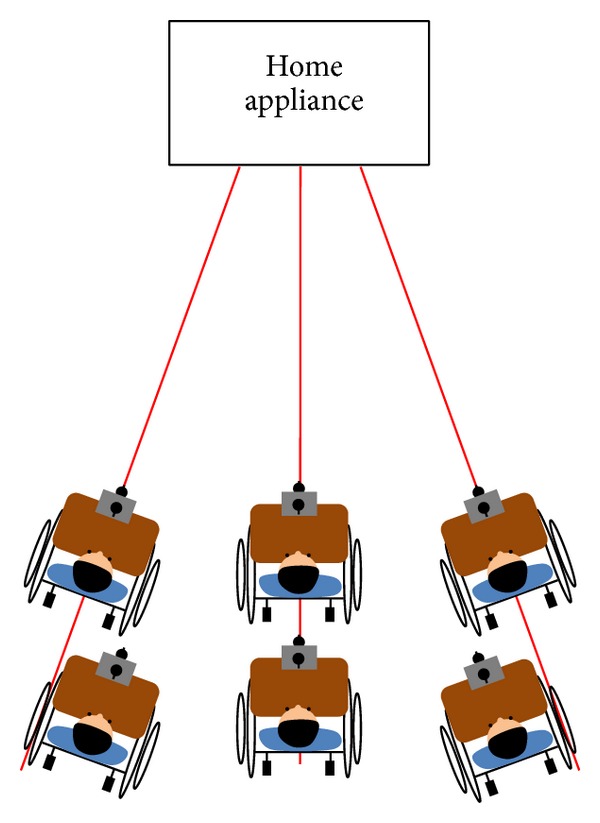
Top view of a sample experimental setup.

Generally, good results cannot be easily obtained for positions close to the borders of an appliance when compared with positions clearly within or outside the borders. In addition, the four positions outside each appliance cannot be easily indicated. Therefore, the following schemes were used in the experiments. In the case when gazing at a home appliance, each user was instructed to randomly look at four positions within the borders of the home appliance with the following instruction: “just look at any four positions inside the home appliance.” In the case when not gazing at a home appliance, the user was asked to randomly look at four positions outside the borders of the appliance with the following instruction: “just look at any four positions close to the home appliance (on the left- and right-hand side and above and below the boundaries of the home appliance).” This procedure of looking at eight positions is one task, and each participant repeated the task five times. Three home appliances were used in the experiments (60-inch television, heater, and air conditioner switch) from three viewpoints (left, center, and right) and two *Z*-distances (television and heater: 270 cm and 300 cm, air conditioner switch: 170 cm and 200 cm), as shown in Figure 15. The angles between the left and center (and the right and center) viewpoints are approximately 20° and 18° at *Z*-distances of 270 cm and 300 cm, respectively. Therefore, 21,600 eye images and 21,600 scene images were obtained for the experiments.

**Figure 16:**
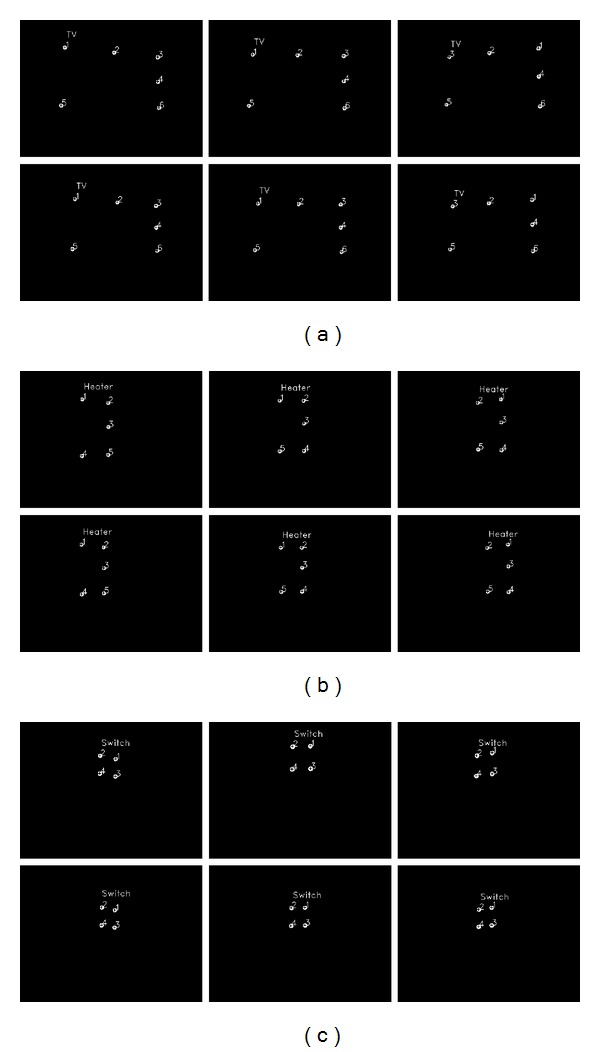
Images of (a) 60-inch television, (b) heater, and (c) air conditioner switch produced by the scene camera with different viewpoints and *Z*-distances (note that the 1st and 2nd row images of (a)–(c) indicate the near and far *Z*-distances, resp., and the 1st, 2nd, and 3rd column images of (a)–(c) indicate the left, center, and right viewpoints, resp.).

Figure 16 shows the images of the different home appliances produced by the scene camera at different viewpoints and *Z*-distances. The upper and lower row images of (a), (b), and (c) of Figure 16 look similar because the difference between the near and far *Z*-distances (the upper and lower row images of (a), (b), and (c) of Figure 16, resp.) is minimal.

The accuracy of selecting one of the three home appliances was calculated using only the pupil center (without compensation with the corneal SR) and using both the pupil centers and the corneal SR (with compensation). Henceforth, these cases will be referred to as gaze estimation methods 1 and 2, respectively.

The accuracy of gaze estimation methods 1 and 2 was quantitatively measured in terms of true positive rate (TPR) and true negative rate (TNR). True positive rate is the rate at which the calculated gaze position is located in the region inside the home appliance boundary when the user is actually gazing at the home appliance. The TNR is the rate at which the calculated gaze position is in the region outside the home appliance boundary when the user is not gazing at the home appliance.

**Figure 17:**
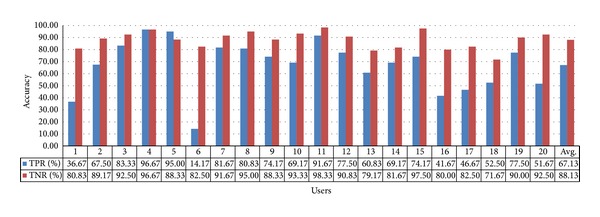
Accuracy of selecting the 60-inch television using gaze estimation method 1.

For the 60-inch television, the TPR and TNR values are 67.13% and 88.13%, respectively, with gaze estimation method 1, as shown in Figure 17. The average value of TPR and TNR is approximately 77.63%.

**Figure 18:**
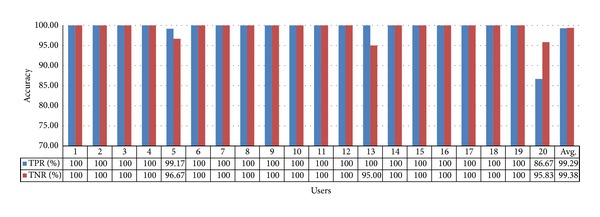
Accuracy of selecting the 60-inch television using gaze estimation method 2.

For this appliance, the TPR and TNR are 99.29% and 99.38%, respectively, with gaze estimation method 2, as shown in Figure 18. The average value of TPR and TNR is approximately 99.34%. By comparing with Figure 17, we can see that the accuracy of gaze estimation method 2 is higher than that of gaze estimation method 1. The TPR and TNR of the 20th user in Figure 18 are the lowest because of incorrect corneal SR detection resulting from an elongated SR, which causes the presence of SRs at the boundary of the iris and the sclera.

**Figure 19:**
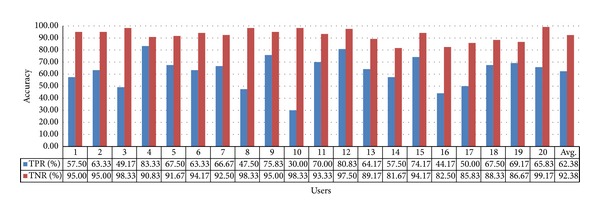
Accuracy of selecting the heater using gaze estimation method 1.

For the heater, the TPR and TNR values are 62.38% and 92.38%, respectively, with gaze estimation method 1, as shown in Figure 19. The average value of TPR and TNR is approximately 77.38%.

**Figure 20:**
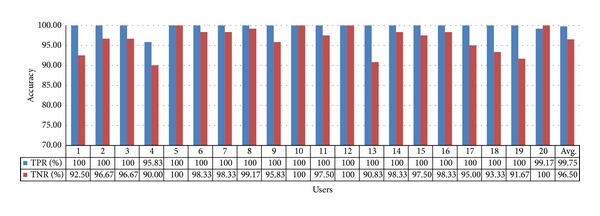
Accuracy of selecting the heater using gaze estimation method 2.

In the experiment with the heater, the obtained TPR and TNR values are 99.75% and 96.50%, respectively, with gaze estimation method 2, as shown in Figure 20. Their average value is approximately 98.13%. The accuracy of gaze estimation method 2 is clearly higher than that of gaze estimation method 1. The TPR and TNR of the 4th user are the lowest because the user did not correctly gaze at the corners of the heater during the initial calibration.

**Figure 21:**
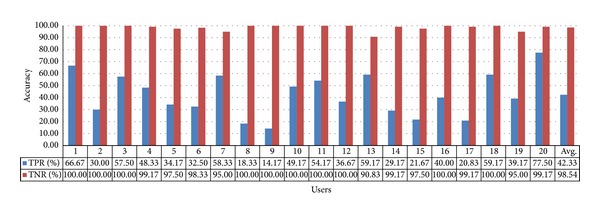
Accuracy of selecting the air conditioner switch using gaze estimation method 1.

In the case of selecting the air conditioner switch, the calculated TPR and TNR values are 42.33% and 98.54% using gaze estimation method 1, respectively, as shown in Figure 21. The average value of TPR and TNR is approximately 70.44%, which is the lowest when compared with the other cases. This is because gaze estimation method 1 is significantly affected by head movements owing to the absence of compensation of the pupil center position by the corneal SR. Because the switch area is smaller than those of the TV and the heater, even small head movements significantly affect the gaze estimation position.

**Figure 22:**
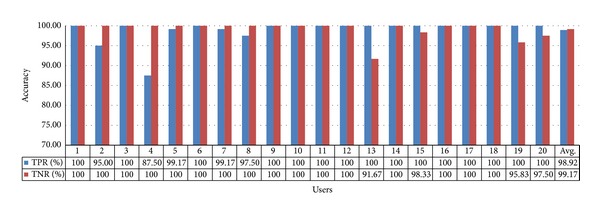
Accuracy of selecting the air conditioner switch using gaze estimation method 2.

For this appliance, the TPR and TNR values obtained using gaze estimation method 2 are 98.92% and 99.17%, respectively, as shown in Figure 22, and their average is approximately 99.05%. By comparing with Figure 21, we can see that the accuracy of gaze estimation method 2 is higher than that of gaze estimation method 1. The TPR of the 4th user and TNR of the 13th user are the lowest because these users did not gaze at the correct corner positions of the switch during the initial calibration. By analyzing the images of pupil center and corneal SR position detection, we can confirm that all the detections are accurate. Because no other factor causes the low accuracy of TPR and TNR, we estimate that the 4th and 13th users of Figure 22 did not gaze at the correct corner positions of the switch during the initial calibration although the positions which they actually looked at are not difficult to be known. In addition, to analyze the effect of initial calibration on the accuracy, the results of these subjects (the 4th and 13th users of Figure 22) were not excluded.

Table 3 summarizes the results shown in Figures 17–22, and the superiority of gaze estimation method 2 can be clearly seen.

### 3.3. Mode Transition of Our System

**Figure 23:**
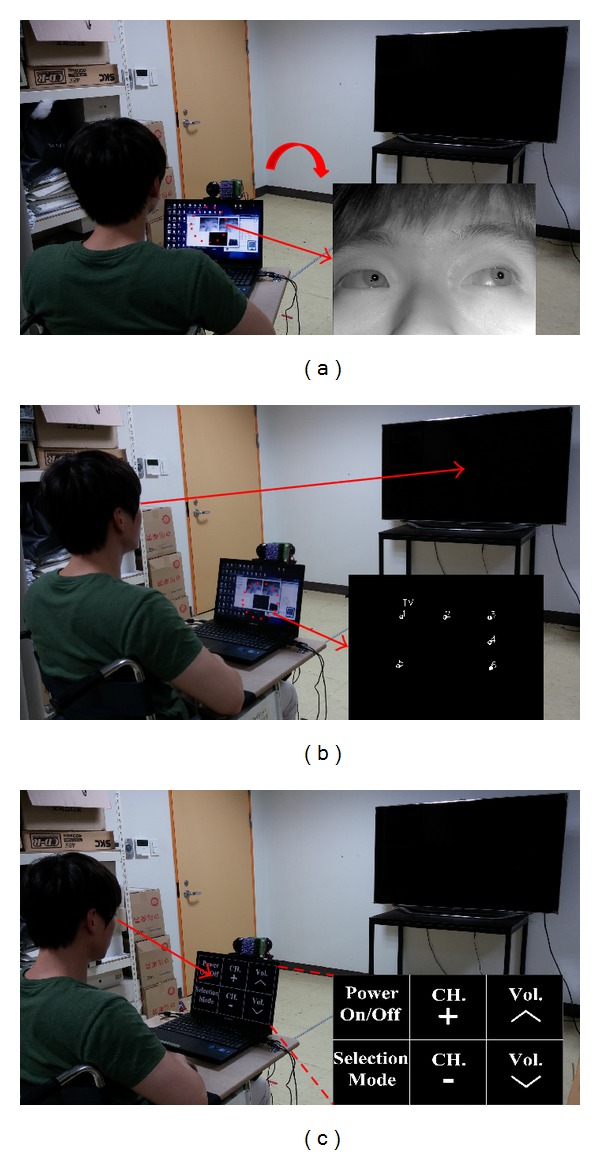
Operation of the proposed system. (a) Navigation mode for moving the wheelchair. (b) Selection mode for determining the home appliance to be controlled. (c) Manipulation mode for controlling the selected home appliance.

In our system, the signal for moving the wheelchair can be triggered according to the direction of gaze movement, as shown in Figure 23(a). If the gaze position is moved in the left- or right-hand side direction, a signal is triggered for rotating the wheelchair in the left- or right-hand side direction, respectively. If the gaze position is moved in the upper or lower direction, a signal is triggered for moving the wheelchair forward or backward, respectively. If the gaze position is at the central area of scene camera image, a signal is triggered for stopping the movement of the wheelchair. As the future work of adopting the electrical motor on the wheelchair or interfacing with electric wheelchair, the user can reorient the wheelchair toward the appliance of interest.

**Figure 24:**
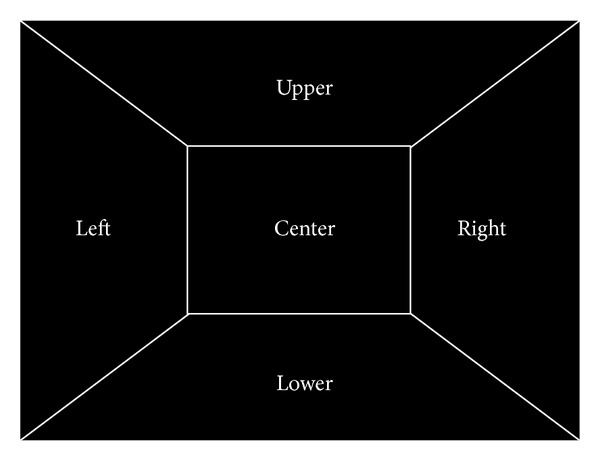
Five areas in the image of frontal viewing camera for discriminating the user's gaze direction in the navigation mode.

In order to evaluate the reliability of our system, the additional experiments of five persons were performed. Each person tried to move the direction of gaze position in the five directions (left, right, upper, and lower directions and central area, resp., as shown in Figure 24). This procedure was iterated five times per each person. Because it is difficult to indicate the reference position to be gazed in 3D space, we told only the instruction to each user, for example, “if you want to rotate the wheelchair in the left-hand side direction, you should move your gaze position in left direction. Please, move your gaze position in the left direction.” Another example is “if you want to move the wheelchair backward, you should move your gaze position in lower direction. Please, move your gaze position in the lower direction.” In order to discriminate the user's gaze direction, we define the five areas in the image of frontal viewing camera as shown in Figure 24.

Accuracy was measured as correct recognition rate which is calculated by the ratio of the number of correctly recognized trials to that of total trials. For example, if four trials for moving the gaze position in the left direction are correctly recognized among total five trials, the correct recognition rate is 80% (100 × 4/5 (%)). Experimental results showed that, for each user and for each trial, all the five gaze directions of the navigation mode were recognized correctly with the accuracy of 100%.

As shown in Figure 23(c), after the specific home appliance is selected by gazing at it for more than 2 s, the control panel with the 3 × 2 menu options is displayed on the laptop computer placed below the gaze tracking system. If the TV is selected, the 3 × 2 menu options are power on/off, selection mode, channel up/down, and volume up/down, as shown in Figure 23(c). If the selected home appliance is the heater or the air conditioner switch, the 3 × 2 menu options are power on/off, selection mode, air direction up/down, and air volume up/down. The user gazes at one of the menu options for a specific time threshold, which can be manually adjusted depending on the user, and the signal for controlling the home appliance is triggered. As the future work of generating the control signal of near-infrared (NIR) light using the communication board such as Arduino board [33], we can actually control the selected home appliance.

In order to evaluate the reliability of our system, the additional experiments of five persons were performed. Each person tried to gaze at six menus five times. We told only the instruction to each user, for example, “just, look at one of the six menus one by one.”

Accuracy was measured as correct detection rate which is calculated by the ratio of the number of correctly detected trials to that of total trials. For example, if three trials for gazing at the menu are correctly detected among total five trials, the correct detection rate is 60% (100 × 3/5 (%)). Experimental results are shown in Table 4, and we can confirm that the reliability of our system in manipulation mode is very high. Because the number of menus of Figure 23(c) are larger than that of regions of Figure 24, and there is large possibility that the pupil is occluded by eyelid or eyelash by gazing at the menu in the downward direction of Figure 23(c), the accuracies of Table 4 are lower than those of navigation mode.

**Figure 25:**
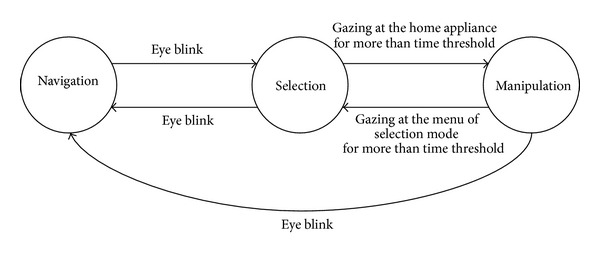
Overall process of transitions between the three modes.

Figure 25 shows the overall process of transitions between the three modes implemented in our system: navigation (moving the wheelchair in the direction of gaze movement), selection (selecting the specific appliance by gaze estimation), and manipulation (controlling the selected appliance by gazing at the control panel).

The transitions (from navigation to selection, from selection to navigation, and from manipulation to navigation) are done by eye closing within a specific time period. The eye closure and openness is detected with the number of black pixels within the detected eye region. The transition (from manipulation to selection) is done by gazing at the one menu of selection mode for a specific time threshold as shown in Figure 23(c). The transition (from selection to manipulation) is done by gazing at the specific home appliance for more than the time threshold. Because the kinds of the 3 × 2 menu options of the control panel to be displayed can be determined after the specific home appliance is selected, there is no direct transition (from navigation to manipulation), and it requires the intermediate transition to selection inevitably.

In order to evaluate the reliability of our transition system, the additional experiments of five persons were performed. Each person tried to perform transitions (from navigation to selection, from selection to navigation, from selection to manipulation, from manipulation to selection, and from manipulation to navigation) five times by eye blink or gazing according to our instruction.

Accuracy was measured as correct detection rate of transition which is calculated by the ratio of the number of correctly detected trials to that of total trials. For example, if four trials for performing transition are correctly detected among total five trials, the correct detection rate is 80% (100 × 4/5 (%)). Experimental results are shown in Table 5, and we can confirm that the reliability of our transition system of modes is very high.

In the initial calibration stage, each user looks at the four corners of a home appliance once, and an additional calibration stage is not required. A home appliance can be selected by looking at it for more than 2 s (this time threshold can be manually adjusted by the user) without additional calibration.

### 3.4. Accuracy with People Imitating Involuntary Head Movements and Scalability of Our System

**Figure 26:**
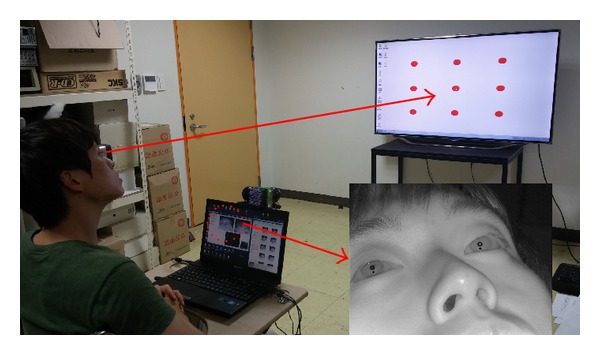
Example of operation of the proposed system with the user imitating involuntary head movement.

It is difficult for us to have actual experiments with cerebral palsy patients, who suffer from involuntary head movements, because of the difficulty involved in collecting the patients under the approval of the institutional review board (IRB). Therefore, the experiments were conducted with two healthy persons who imitated involuntary head movements, as shown in Figure 26. The diameter of each reference point is 6 cm. Experimental results showed that the gaze estimation uncertainty (error) was ±2.4° with the standard deviation of 1.99. Although this error was higher than that without involuntary head movements of Table 2, we can find that the navigation, selection, and manipulation process of Figure 25 can be successfully performed by our system. And we can expect that our system can be used by people suffering from involuntary head movements.

**Figure 27:**
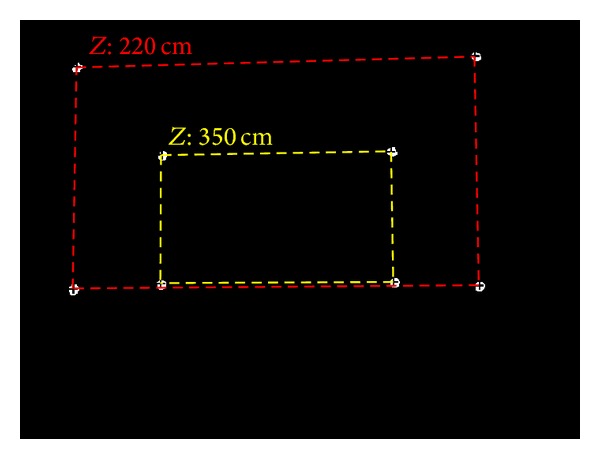
Variation in rectangle size with *Z*-distances.

The upper and lower row images of (a), (b), and (c) of Figure 16 look similar because the difference between near and far *Z*-distances (the upper and lower row images of (a), (b), and (c) of Figure 16, resp.) is not large. In the case of the television and the heater, the near and far *Z*-distances are 270 cm and 300 cm, respectively. In the case of the air conditioner switch, they are 170 cm and 200 cm, respectively. As shown in Figure 27, the size of the rectangle formed by the four NIR LED spots at a far *Z*-distance of 350 cm is much smaller than that at near *Z*-distance of 220 cm because the difference between the near and far *Z*-distances is significant.

**Figure 28:**
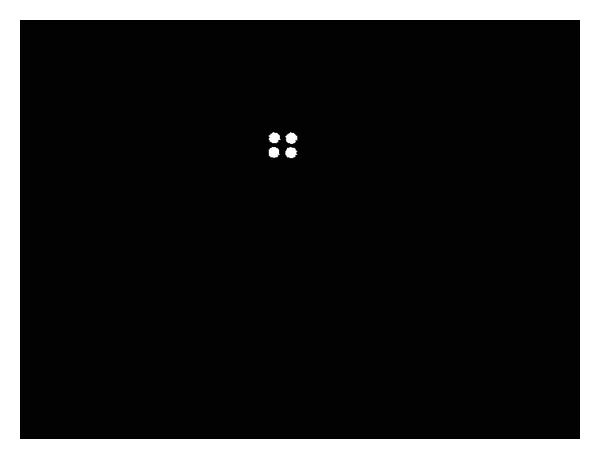
Frontal image of air conditioner switch at a *Z*-distance of 375 cm.

As shown in Figure 16, different NIR LED patterns can be produced using different quantities and positions of NIR LEDs. Therefore, many home appliances can be recognized, and consequently the scalability of our approach is high. However, when the distance between two NIR LED spots is minimal because of the longer *Z*-distance between the camera and appliance (if the *Z*-distance is greater than the case of Figure 28 with the air conditioner switch at the *Z*-distance of 375 cm), the NIR LED spots cannot be easily located; thus, the home appliance cannot be recognized. However, the case where a user would want to select an air conditioner switch at a far *Z*-distance considering typical eyesight and typical home size is a rare event. Therefore, the proposed method is highly scalable, and numerous home appliances can be recognized using our method.

## 4. Conclusion

In this study, we proposed a novel interface system consisting of nonwearable eye tracking and scene cameras to select and control home appliances. The performance of the proposed system was investigated with three home appliances at different positions and *Z*-distances. In addition, the performance of two gaze-estimation methods was evaluated. Because the proposed system enables users to control home appliances by looking at them, instead of using a display-based menu, this approach might be more natural and convenient for users.

In the future, we would like to study the recognition of multiple home appliances in images captured by a scene camera. Additionally, we will study methods of enhancing the accuracy of 3D gaze estimation by considering the *z*-axis in addition to the *x*- and *y*-axes.

## Figures and Tables

**Table 1:** Summaries of proposed and previous approaches.

Category			Method	Advantages	Disadvantage
Gaze tracking on 2D screen	Wearable device [9, 10, 15]		Eye images are acquired by wearable gaze tracking device.	The accuracy of gaze estimation is usually higher than that by nonwearable device.	Application is restricted to interaction with a 2D screen.
	Nonwearable device [11–14]		Eye or face images are acquired by nonwearable gaze tracking device.	User convenience is higher than that by wearable device.	

Gaze tracking in 3D space	Wearable device [16–21]		Scene and eye images are acquired by wearable device.	The allowable range of head rotation is large, because the wearable gaze tracking device is moved according to head movement.	User inconvenience increases by wearing the device including two cameras for gaze estimation and frontal viewing.
	Nonwearable device	Frontal viewing camera sensing visible light [16]	Scene and eye images are acquired by nonwearable devices.	User convenience is higher than that by wearable device.	Complicated algorithm is required to recognize the object in the visible light image given by frontal viewing camera.
		Frontal viewing camera sensing NIR light (**proposed method**)		The object in the frontal viewing image can be easily detected and recognized using the NIR camera and NIR LED pattern.	Additional NIR LEDs must be attached to the outer boundary of the object to be recognized.

**Table 2:** Gaze estimation uncertainties in proposed system.

User	Error (standard deviation)
User 1	±0.91° (0.33)
User 2	±0.75° (0.08)
User 3	±0.68° (0.09)
User 4	±2.03° (0.37)
User 5	±2.06° (0.24)
User 6	±0.77° (0.16)
User 7	±0.96° (0.14)
User 8	±0.51° (0.14)
User 9	±0.92° (0.13)
User 10	±0.79° (0.12)

Average	±1.04° (0.18)

**Table 3:** TPR and TNR values (and standard deviation) of the three home appliances using gaze estimation methods 1 and 2 (%).

Home appliance	Accuracy	Gaze estimation method 1	Gaze estimation method 2
60-inch television	TPR (std.)	67.13 (0.21)	99.29 (0.03)
	TNR (std.)	88.13 (0.07)	99.38 (0.02)
	Avg. (std.)	77.63 (0.14)	99.34 (0.02)

Heater	TPR (std.)	62.38 (0.13)	99.75 (0.01)
	TNR (std.)	92.38 (0.05)	96.50 (0.03)
	Avg. (std.)	77.38 (0.09)	98.13 (0.02)

Switch for air conditioner	TPR (std.)	42.33 (0.18)	98.92 (0.03)
	TNR (std.)	98.54 (0.02)	99.17 (0.02)
	Avg. (std.)	70.44 (0.10)	99.05 (0.03)

Avg. (std.)		75.15 (0.11)	98.84 (0.02)

**Table 4:** Accuracies of trials of manipulation mode (%).

User	Menu positions						Average
	Upper-left	Upper-center	Upper-right	Lower-left	Lower-center	Lower-right	
User 1	60	80	100	100	100	100	90
User 2	80	100	100	100	100	100	96.67
User 3	80	100	100	100	100	100	96.67
User 4	80	100	80	100	100	100	93.33
User 5	100	100	100	80	100	80	93.33

Average	80	96	96	96	100	96	94

**Table 5:** Accuracies of trials of transitions between modes (%).

User	Transitions					Average
	Navigation to selection	Selection to navigation	Selection to manipulation	Manipulation to selection	Manipulation to navigation	
User 1	100	80	100	100	100	96
User 2	100	100	100	100	100	100
User 3	100	80	100	100	80	92
User 4	80	100	100	100	100	96
User 5	100	100	100	80	80	92

Average	96	92	100	96	92	95.2
